# Covid-19 infection risk on US domestic airlines

**DOI:** 10.1007/s10729-022-09603-6

**Published:** 2022-07-02

**Authors:** Arnold Barnett, Keith Fleming

**Affiliations:** MIT Sloan School of Management, E62-568, MIT, Cambridge, MA 02142 USA

**Keywords:** Covid-19, Mathematical modeling, Probability, Risk analysis

## Abstract

Working with recent data and research findings, we estimate the probability that an air traveler in economy class would have contracted Covid-19 on a US domestic jet flight over the nine-month period June 2020 to February 2021. The estimates take account of the rates of confirmed Covid-19 infections in the US, flight duration, fraction of seats occupied, and some demographic differences between US air travelers and US citizens as a whole. Based on point estimates, the risk of contracting Covid-19 in-flight exceeded 1 in 1000 on a fully-loaded two-hour flight at the height of the pandemic over the nine months, but was about 1 in 6000 on a half-full flight when the pandemic was at a low ebb. However, these estimates are subject to substantial uncertainty, with the 10th percentiles of various risk distributions only about 1/7 as large as the medians, and the 90th percentiles about four times as large. Based on seat-occupancy levels on US flights for each month over June 2020 to February 2021, the median risk estimate for that period is 1 in 2250, while the mean risk estimate is 1 in 1450. Indirect effects arose because those who contracted Covid-19 on US airplanes could in turn infect others.

**Supplementary Information:**

The online version contains supplementary material available at 10.1007/s10729-022-09603-6.

## **Highlights**

- Focusing on the period June 2020–February 2021, the paper estimates the probability that a randomly-chosen passenger uninfected by Covid-19 contracted the disease during an average (two-hour) domestic jet flight in the United States
- The analysis uses numerous data sets to estimate the probability that a passenger boarding a US domestic flight over the observation period carried contagious Covid-19. That probability varied considerably over the nine months considered.
- In calibrating its probabilistic model, the paper uses data from all available peer-reviewed papers that specified the seating location(s) of passengers who harbored Covid-19 when they boarded a flight, and also the seating locations of other passengers who did and did not contract the coronavirus in flight.
- The point estimate for the probability of contracting Covid-19 on board an average domestic flight was about 1 in 2000 for the nine-month study period. That estimate is subject to sizable uncertainty.
- The approach to modeling advanced in the paper can be adapted to make risk estimates in other circumstances, in connection with both the coronavirus and other infectious diseases.

## Introduction

This paper estimates the probability that, early during the Covid-19 pandemic, a US domestic air traveler would contract the coronavirus while in flight. The focus is on the nine-month period from June 2020 to February 2021, a time frame that excludes both (i) the first months of the pandemic, when US air travel came practically to a halt, and (ii) the period starting in March 2021, when the use of vaccines was accelerating. There was no consensus on the magnitude of in-flight infection risk during those nine months. The nationally-renowned expert Dr. Anthony Fauci stated that he would not fly until the pandemic subsided, while the US Centers for Disease Control and Prevention (CDC) warned against air travel throughout the nine-month period. Yet United Airlines declared that Covid-19 transmission risk was “nearly nonexistent” even on its full flights, while Southwest Airlines characterized the risk as “virtually nonexistent.”

It is not easy to estimate the risk that an uninfected passenger on a US domestic flight would contract Covid-19. Here we attempt to do so with a probabilistic model which assumes that three things must occur for an in-flight infection to arise: there must be at least one contagious passenger on board, universal mask wearing must fail to prevent transmission, and the uninfected traveler must be seated fairly close to a contagious one. (As we will explain, we treat other means of on-board transmission as second-order effects.) The model estimates the joint probability of these three events.

The primary focus here is on economy-class passengers who took two-hour US domestic flights on either Boeing 737 or Airbus 320 jets (two hours is the average duration of domestic flights). We make estimates of Covid-19 infection risk on a month-by-month basis during the observation period, using the actual distribution of seat-occupancy levels in each month. For full flights, our approximate distribution for the probability that a randomly chosen uninfected passenger would contract Covid-19 in flight has a median of 1 in 2250 and a mean of 1 in 1450. The 10th percentile of the estimate is about 1 in 14,000, while the 90th percentile is about 1 in 600.

Below we start with a literature review, followed by development of the general risk model for Covid-19 transmission, then the estimation of model parameters, and then the presentation of risk estimates.

## Some relevant literature

There is a voluminous literature about the transmission of viral infections in closed spaces. Berry et al. [1] summarized much of this work and assessed its general implications about limiting the spread of Covid-19. Noakes et al. [2] modelled the transmission of airborne infections in indoor environments, based on such factors as ventilation rate and room occupancy. Bazant and Bush [3] proposed upper limits on “cumulative exposure time” for uninfected people in an indoor space, given an occupant with contagious Covid-19 who exudes respiratory droplets. . Chen and Liao [4] offered probabilistic analyses about indoor transmission of various influenza viruses, as a function of viral kinetics, aerial transmission potential, and population dynamics . A meta-analysis performed by Chu et al. [5] considered 216 studies about numerous aspects of viral transmission.

Long before the Covid-19 pandemic, the transmission of infectious diseases during passenger flights was recognized as a serious health hazard. Evidence of in-flight infection arose in connection with SARS and the H1N1p flu pandemic [6, 7], and public health agencies including the World Health Organization and US Centers of Disease Control (CDC) concluded that an uninfected traveler is at greatest risk if seated within two rows of a contagious passenger [8, 9].

Modeling viral transmission in airplanes in extremely difficult, but efforts have been made to do so approximately. Infections can spread via respiratory droplets or aerosols, and models of such spread in aircraft have typically focused on one of these modes of transmission. For aerosols alone, several simulations of viral diffusion have been performed based on the Navier-Stokes equation [10–13]. For droplets, the studies include [14–17], but only the first of these is specific to passenger aviation.

Several papers have discussed actual experiences with viral transmissions on airplanes.

[18–27] In this article, we have attempted to locate the full set of peer-reviewed papers with data about transmission of Covid-19 in flight. We then focused on all nine of those papers which reported which seat(s) were occupied by travelers with contagious Covid-19, which nearby seats were occupied, and which nearby travelers contracted Covid-19 during the flight.

Furthermore, much recent research has appeared concerning the course of Covid-19 among individuals infected with it. This research estimates what fraction of carriers are eventually symptomatic [28], the period of contagiousness both before and after symptoms occur [29], the distribution of symptomatic cases by severity [30], and the duration of contagiousness of asymptomatic carriers [31, 32]. These various patterns are germane to estimating the likelihood that a passenger boarding a plane has a contagious case of Covid-19.

## The general model

We focus on Boeing 737 or Airbus 320 jet aircraft in an all-coach configuration with 174 seats, in 29 rows with six seats apiece. In each row, there are two sets of three seats on opposite sides of a center aisle (seats ABC to the left of the aisle, DEF to the right). We consider nonstop US domestic flights of two-hour duration over the nine-month period June 2020–February 2021. The basic equation for estimating the probability P, that a randomly-chosen uninfected traveler contracts Covid-19 in flight is

1 $$P=\sum_{N,X}{Q}_{N,X}{P}_{N,X}$$

where

P: P(randomly-chosen uninfected passenger contracts Covid-19 in flight)
*P*_*N*, *X*_: *P*(randomly − chosen uninfected passenger boards flight with *N* passengers,  X of whom board harboring Covid-19)
*Q*_*N*, *X*_: *P*(randomly chosen uninfected passenger contracts Covid − 19 on board | N, X)

That P depends on X, the number of contagious passengers on board the flight, is obvious. The total number of passengers (N) matters because, the greater the number on board, the larger in general is the *fraction* of travelers seated in close proximity to contagious ones.

Below we first estimate *P*_*N*, *X*_ and then the conditional probability *Q*_*N*, *X*_.

### The distribution of N

Suppose that M is the number of nonstop flights on 737/A320 aircraft (with an estimated 174 seats) in a particular month, and let q_Z_ be the proportion of those flights with exactly z passengers on board. Then the total number of passengers carried on flights with z passengers would be Mzq_z_, and the fraction Q_z_ of all travelers that month aboard such flights would follow:

2 $${Q}_z=P\left(N=z\right)=\kern0.5em Mz{q}_z/\sum_{j=0}^{174} Mj{q}_j=\frac{z{q}_z}{\sum_{j=0}^{174}j{q}_j}$$

In practice, we work with data for small ranges of z rather than individual values. As we will discuss, we had access to data about seat-occupancy rates by month on US aircraft between June 2020 and February 2021.

### The distribution of X

For a particular value of N and a particular day, let ***p***_***CONTAG***_ be the probability that a randomly-chosen passenger boarding one of the flights under study carries contagious Covid-19. We treat different passengers as independent in terms of contagiousness status, meaning that X, the number of contagious travelers on board, would follow a binomial distribution with parameters N and *p*_*CONTAG*_ . Estimating *p*_*CONTAG*_ is therefore the key to approximating the distribution of X.

Our general strategy for estimating ***p***_***CONTAG***_ is:

- Estimate the fraction of Americans who carry Covid-19 at the time of the flight
- Adjust that fraction to reflect the fact that air travelers are more affluent than average Americans, and the Covid-19 infection rate is negatively correlated with income
- Further adjust that fraction to reflect the likelihood that carrying Covid-19 reduces the likelihood of actually boarding a plane. Take into account that this statement is least true for those who are asymptomatic/pre-symptomatic, and most true for those with serious/critical cases.

### The fraction γ of potential air passengers who carry Covid-19

To estimate ***p***_***CONTAG***_, we first estimate the fraction of potential US air travelers who carry Covid-19 at a given time. The general equation for *γ* is:

3 $$\gamma =\left(\frac{C_7}{POP}\right)\rho \beta .$$

Where

*C*_7_: Number of confirmed cases of Covid − 19 in the US over the past seven days
*POP*: Population of the US
*ρ*: a multiplier to adjust for the (sizable)extent to which confirmed Covid − 19 cases in the US underestimate the actual number of cases
*β*: a^"^healthy passenger^"^ factor to reflect the fact that US air travelers come disproportionately from communities where Covid − 19 is less common than in the population at large.

The quantity C_7_ enters (3) because seven days is approximately the duration of contagiousness for a carrier of Covid-19 [29, 32]. Thus, based on *confirmed* cases, $\frac{C_7}{POP}$ approximates the fraction of Americans who are contagious at a particular time. Because we are considering US domestic flights as a whole, it seems reasonable to work with national statistics about new infections.

However, a substantial share of new cases of Covid-19 do not get confirmed in the US, meaning that actual cases over a seven-day period considerably exceed confirmed cases. There is no official guidance in the US about the ratio of actual-to-confirmed new cases at any time.. Here the quantity *ρ* is an estimate of that ratio, which we approximate on a weekly basis over the nine-month observation period.

### Underestimation of US Covid-19 cases (the multiplier ρ for confirmed cases)

To estimate *ρ*, we follow a process of “reverse engineering,” using death counts at the particular times to estimate numbers of new cases at previous times. Suppose that *η* is the fraction of Covid-19 cases end in death. Thus, having estimated how many cases that started at a given time ultimately caused deaths, we could multiply by 1/*η* to approximate the total number of new cases at that time.

We made the approximation based on literature that those who died on a given date contracted their fatal cases of Covid-19 about uniformly over a four-week period that ended fourteen days earlier [30]. Thus, for new cases on day t that ultimately ended in death, these deaths occur in about equal numbers on each day from t + 14 to t + 41. However, on (say) day.

t + 18, there would also be deaths resulting from new cases on days t – 23 to t + 4. We estimate the number of new infections on day t that turned fatal under the rule:

4 $${N}_D(t)\kern0.5em \approx \left(\frac{1}{28}\right)\kern0.5em \sum_{j=14}^{41}D\left(t+j\right)$$

Where

*N*_*D*_(*t*): Number of new US Covid − 19 cases on day t that ended in death
*D*(*t* + *j*): recorded number of US Covid − 19 deaths on day t + j

In essence, (4) says that because day t was one of 28 contributors to the death toll on each day from t + 14 to t + 41, it contributed about 1/28 of the total deaths of that period. On average, that rule seems plausible.

Once *N*_*D*_(*t*) is at hand, it would be scaled up by the aforementioned factor 1/*η* to approximate the total number of new cases on day t. *η* would be based on estimates in the literature on the fraction of US Covid-19 cases over June 2020–February 2021 that ended in death. *ρ* would then be approximated by the ratio of the number of new cases based on death counts to the number of confirmed cases (C_7_):

5 $$\rho \approx \left(\frac{1}{\eta}\right)\frac{N_D(t)}{C_7}$$

### “Healthy traveler” factor β

We consider a region that is divided into various communities, and contrast the overall Covid-19 rate for the region with a rate that weights individual communities by their shares of air travelers. The estimation uses four quantities for each community for 2020: its population, its median income, its per-capita rates of confirmed Covid-19 infections, and the per-capita annual rates at which its residents took air trips (before the Covid-19 pandemic).

If the communities are then combined into n ranges based their median incomes, the chance that a randomly-selected citizen has experienced Covid-19 can be approximated by:

$${R}_{CV}=\sum_{i=1}^n{R}_i{I}_i$$

where

*R*_*CV*_: probability of having had Covid-19.
*R*_*i*_: probability of having had Covid − 19 for citizens of communities in median −income range i.
I_i_: fraction of all citizens living in communities in median − income range i
(n): number of median − income ranges

Focusing on air travelers rather than randomly-chosen citizens, suppose that *T*_*i*_ = annual air trips per capita in communities in median − income range i

Then ${T}_i{I}_i/\sum_{j=1}^n{T}_j{I}_j$ is the fraction of pre-pandemic air trips taken by people from communities in median-income range i. If we weight each range’s per-capita Covid-19 rates by shares of pre-pandemic air trips, we reach an adjusted risk level *R*_*CVT*_ that is given by:

$${R}_{CVT}=\sum_{i=1}^n{R}_i{T}_i{I}_i/\sum_{j=1}^n{T}_j{I}_j$$

*R*_*CVT*_ is the probability of having had Covid-19, giving greater weight to communities in the income ranges with disproportionate shares of air travelers.

An estimate of the “healthy traveler” factor *β* would be:

6 $$\beta \approx {R}_{CV T}/{R}_{CV}$$

As we will discuss, we calculated a *β*−estimate with data from the Commonwealth of Massachusetts, and made another estimate using data from England and Wales.

This approximation for *β* is not perfect. It treats the pre-Covid distribution of air trips by income as a good proxy for the distribution during the Covid-19 pandemic, and bases trips per capita in a given community on its median income rather than its distribution of income. Avoiding such assumptions is not feasible given the limitations of available data.

$\mathrm{As}\ \mathrm{noted}\ \mathrm{earlier},\gamma =\left(\frac{C_7}{POP}\right)\rho \beta \kern0.75em$ However, *γ* is not in itself the probability ***p***_***CONTAG***_ that we seek**,** as we explain below.

### The probability ***p***_***CONTAG***_ that a boarding air passenger carries Covid-19

Suppose that *α* is the probability that a potential air traveler *not* suffering from Covid-19 would fly on a given day during the pandemic. (This quantity takes account of the general reluctance to travel because of the pandemic.) Among those who carry Covid-19—the number of which depends on *γ*−we posit that their chance of flying is *α* times a filtering parameter *f* that is less than one. The presumption is that some disease carriers refrain from flying because they suspect or know that they are infected with Covid-19 or simply feel too ill to travel.

We treat f as a function of several other quantities. An individual who carries Covid-19 who might potentially fly would fall into one of four categories:

**Table:** 

Category	P(someone carrying Covid-19 is in that Category)
Asymptomatic carrier	*P*_*ASYM*_
Pre-symptomatic carrier	*P*_*PRESYM*_
Carrier with mild/moderate symptoms	*P*_*MS*_
Carrier with severe/critical symptoms	*P*_*SC*_

In Section 4, we will estimate these probabilities based on medical literature.

We define three quantities related to potential travelers who harbor Covid-19

*ψ*_*NS*_: the probability that someone with asymptomatic/pre-symptomatic Covid-19 who would otherwise fly will *not* do so
*ψ*_*MS*_ : the corresponding probability for someone showing mild/moderate symptoms of Covid-19
*ψ*_*CS*_: the corresponding probability for someone showing severe/critical symptoms of Covid-19

The quantities *ψ*_*NS*_, *ψ*_*MS*_, *and ψ*_*CS*_  *ψ*_*NS*_ are “desistance” probabilities, which reflect an unwillingness to fly among Covid-19 carriers beyond the general level of unwillingness signified by the parameter *α*. *ψ*_*NS*_ is relevant because someone with asymptomatic/pre-symptomatic Covid-19 might not fly because he knows or suspects that he carries the virus. *ψ*_*MS*_ is the chance that someone with mild/moderate symptoms foregoes the flight, and *ψ*_*CS*_ is the chance for severe/critical carriers. One can reasonably postulate that *ψ*_*NS*_ < *ψ*_*MS*_< *ψ*_*CS*_, but assigning probability distributions to *ψ*_*NS*_, *ψ*_*MS*_, *and ψ*_*CS*_ is not easy.

If *αf* is the probability a potential air traveler who carries Covid-19 nonetheless flies, the filter f would follow:

7 $$f=\left({P}_{ASYM}+{P}_{PRESYM}\ \right)\left(1-{\psi}_{NS}\right)+\kern0.75em {P}_{MS}\left(1-{\psi}_{MS}\right)+{P}_{SC}\left(1-{\psi}_{CS}\right)$$

The conditional probability ***p***_***CONTAG***_ that a passenger who boards a flight carries Covid-19 would then follow:

8 $${\boldsymbol{p}}_{\boldsymbol{CONTAG}}=\frac{\alpha f\gamma}{\alpha f\gamma +\alpha \left(1-\gamma \right)}=\frac{f\gamma}{f\gamma +\left(1-\gamma \right)}$$

where *γ* and f arise from (3) and (7)

Combining the various elements above, we reach:

9 $${P}_{N=j,X=k}\approx \kern0.5em P\left(N=j\right)\ast P\left(X=k\ \right|\ X\ is\ B\left(N,{\boldsymbol{p}}_{\boldsymbol{CONTAG}}\right)\Big)$$

where P(N = j) arises from (2), and *B*(*N*, *q*)*i*s binomial with parameters *N* and *q*)

### Estimation of Q_N,X_

Given that there are N passengers on board, X of whom carry Covid-19, Q_N,X_ is the conditional probability that a random-chosen passenger from among the uninfected N – X will contract Covid-19 during the flight. We consider first the case in which X = 1, with a contagious passenger who is seated in seat S_1_.

For an uninfected in another seat S_2_, we posit that the probability of an on-board infection depends on:

- The proximity of S_2_ to S_1_, based on both distance and the presence of any transmission barriers between the seats
- The duration of the flight
- The failure rate of universal masking

We further assume that short interactions among passengers during boarding, deplaning, or en route to the lavatory present second-order risks relative to those of sitting for hours close to a contagious person. We do so because CDC advised that someone exposed to a person with confirmed Covid-19 need not go into quarantine if the interaction was shorter than 15 minutes If, however, one believes that these hazards are not negligible, they would add to the risks we are estimating here.

Directly estimating the Covid-19 transmission probability on aircraft is very difficult. It depends on the contagious passenger’s emissions of the virus via a mixture of breathing, speaking, coughing or sneezing, as well as on the movement of droplets and aerosols given the geometry of the jet airplane and its powerful HEPA air-purification systems. In modeling Q_N,X,_ we lean heavily on two influential journal articles:

- a dynamic-network simulation of viral transmission on US jet aircraft which appeared in the *Proceedings the National Academy of Sciences* [14] It assumes a fixed risk *per minute* that contagious passenger infects a given noninfected passenger within one meter (with a point estimate of 1.8% per minute)
- a meta-analysis which appeared in The Lancet [5], and estimated that, absent barriers and masks, risk of viral infection (per minute) drops off exponentially with distance, at roughly a *factor of two* per meter.

Our general equation for estimating risk *per minute* given S_1_ and S_2_ takes the form:

10 $${V}_{S_{1,}{S}_2}={\pi}_{0}{e}^{-\omega d}{\left(1-\lambda \right)}^R\left(1-{p}_{masks}\right)$$

Where

d: grid distance between contagious and given uninfected passenger
*ω*: rate of exponential decay of risk with distance assuming no barriers
*λ*: P(an individual seatback blocks transmission from a contagious passenger)
*π*_0_: level of risk at distance zero from contagious person
R: number of rows between contagious and uninfected passengers
p_masks_: P(universal masking blocks the transmission of Covid-19 from a contagious passenger to an uninfected one)

We amplify on (10) below.

In measuring d, we use not the Euclidean distance between S_1_ and S_2_ but instead the “grid distance” (i.e., d((x_1_, y_1_) to (x_2,_ y_2_)) = |x_2_ – x_1_| + |y_2_ – y_1_|). This choice reflects the assumption that emissions from a contagious traveler in 16A that reach the breathing space of a passenger in 15B largely travel there via the breathing space for the passenger in 15A.

In including the quantity *λ in* (10), we recognize that the seatback directly ahead of a disease sufferer can block some forward transmission of aerosols and droplets, while that person’s own seatback can somewhat protect passengers one row behind. There is apparently no literature about the health benefits of airplane seatbacks, though an article in a medical journal informed potential air travelers that “seat backs provide a partial physical barrier [33].) We define *λ* as the probability that a single row of seatbacks prevents transmission, in which case 1 - *λ* is the probability that it fails to do so. If there are R rows separating S_1_ from S_2_, we assume that the overall failure risk is (1 − *λ*)^*R*^ because R rows of seatbacks all must fail for transmission to occur. R = 0 if both S_1_ and S_2_ are in the same row.

The model in (9) follows [14] in assuming a constant infection risk per minute assuming no prior transmission, and follows [5] in assuming exponential decay of risk with distance. In the next section, we estimate the parameters *π*_0_, *ω*, *and p*_*masks*_ based on the literature, assigning them probability distributions as well as point estimates.

For a flight of duration T in minutes, the probability ${Z}_{S_1,{S}_2}$ of Covid-19 transmission conditioned on S_1_ and S_2_ follows:

11 $${Z}_{S_1,{S}_2}=1-{\left(1-{V}_{S_{1,}{S}_2}\ \right)}^T$$

If N passengers occupy N seats aboard a flight, we assume that the single contagious passenger is equally likely to be in any of those seats while a randomly-chosen uninfected passenger is equally likely to be in any of the other N – 1 seats. Therefore, *U*(*S*_1_), the probability conditioned on S_1_ that a random uninfected passenger contracts Covid-19 on board would be given by:

$$U\left({S}_1\right)=\left(\frac{1}{N-1}\right)\sum_{all\ {S}_2\ne {S}_1}\ {Z}_{S_1,{S}_2}$$

The overall risk of infection *Q*_*N*, *X* = 1_ would follow:

12 $${Q}_{N,X=1}=\left(\frac{1}{N}\right)\sum_{S_1= Seat\ 1}^{Seat\ N}U\left({S}_1\right)$$

where the seats 1 through N are numbered under some convention

If N = 174 on the 737/A320 jet under consideration, then all seats are full. As N drops below 174, we assume that as many of the empty seats as possible are center seats.

Given that *Q*_*N*, *X* = 1_is small (as we will see), the risk of contracting Covid-19 in flight can be treated as essentially linear in the number of contagious people who board. Thus, based on (12) we can write

13 $${Q}_{N,X=k}\kern0.75em \approx \kern1.25em \left(\frac{k}{N}\right)\sum_{S_1= Seat\ 1}^{Seat\ N}U\left({S}_1\right)$$

Given (1), (9), and (13) , we have an estimate of P.

## Probability distribution for key parameters

Here we pursue the general approaches just outlined to get probability distributions for each model parameter. These distributions will underpin the simulation analysis in the next section.

### Distribution of ρ, (actual Covid-19 cases/confirmed cases)

The “reverse engineering” procedure depicted by eq. (4) generates estimates of the average daily number of new *fatal US* cases of Covid-19 from June 2020 to February 2021. To reach a total number of new cases, one needs divide new fatal cases by the fraction of infections that lead to death (the case fatality rate). That fraction is not precisely known. Baud et al. estimated in June 2020 that 0.7% of Covid-19 infections in the US ended in death [34]. Improvements in treatment for Covid-19 are believed to have lowered the case fatality rate during 2020 by perhaps 20–33%, to about 0.5% [35]. Ioannidis classified the US among nations with a case fatality rate of 0.57% [36].

**Fig. 1:**
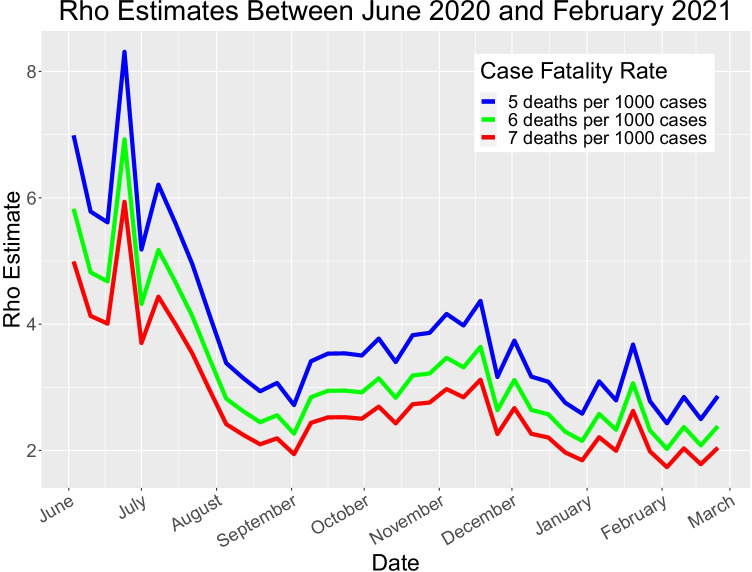
Estimated distribution of ρ, the ratio of new actual US Covid-19 cases to confirmed cases, June 2020–February 2021. Sources: References [34–37]

We estimate *ρ* on a weekly basis to avoid day-of-week variations in confirmed cases. For a point estimate of the ratio *ρ* in a given week, we first estimated total deaths that week using [37]) for each of the seven days. Then we multiplied that quantity by 167 to reach a point estimate of *total* cases, based on a case fatality rate of 0.6% (about the average of the estimates above). We approximated *ρ* as the ratio of total to estimated cases. We assumed (approximately) a normal distribution for *ρ*, treating the point estimate based on a 0.6% fatality rate as its median, the *ρ* − *estimate* based on a 0.7% rate as its 10th percentile, and the estimate based on 0.5% as the 90th percentile. Fig. 1 presents graphically the movement over time of the estimated 10th, 50th, and 90th percentiles for *ρ*.

#### Distribution of $\beta\ \left({}^{"}{\mathsf{Healthy}\ \mathsf{Traveler}}^{"}\kern0.5em \mathrm{effect}\right)$

Table 1 presents data about both confirmed Covid-19 cases and estimated travel frequencies across several income ranges in Massachusetts, which can be processed in accordance with eq. (3). Somewhat surprisingly, the point estimate of *β* arising from the calculations is 0.86, which suggests that the “healthy traveler” effect is not all that large.

**Table 1:** Average annual airline trips and confirmed Covid-19 cases per capita by annual household income, estimates for commonwealth of Massachusetts

Median household income	Average annual flights^1^	Average Covid-19 positive rate (Through 1/9/21)^2^
Below $25,000	0.8	0.066
$25,000-49,999	0.85	0.095
$50,000-74,999	1.65	0.070
$75,000-99,999	4.25	0.042
$100,000-149,999	3.15	0.053
$150,000 and Higher	5.5	0.028

Sources: 1: Airlines for America [38], for US residents over 2016–17, 2: Massachusetts Department of Public Health [39]

For further perspective about *β*,we use data from England [40] about Covid-19 death rates as a function of the deprivation index used to classify small geographic areas. Doing so yields an estimate of *β* for England and Wales of about 0.75.

A reasonable model that incorporates these two estimates and some further uncertainty could posit that *β* follows a beta distribution with parameters *α* = 28 *and β* = 7. This distribution has a mean of 0.8, a slightly higher mode, and a standard deviation of 0.066.

### Distributions for ψ_NS_, ψ_MS_, and ψ_CS_, contributors to the “filtering” effect of carrying Covid-19 on the likelihood of air travel

Assigning probability distributions to the “deterrence” quantities *ψ*_*NS*_, *ψ*_*MS*_, *and ψ*_*CS*_ is difficult, because no readily-available data allow for direct estimates. One can, however, reasonably postulate that *ψ*_*NS*_ < *ψ*_*MS*_< *ψ*_*CS*_, and try to work with scant existing “clues” about the magnitudes of these parameters.

The quantity *ψ*_*NS*_ for carriers without symptoms would presumably be low. Such individuals could well decline to travel if they test positive for Covid-19, or have recently been close to others with known infections. But many US cases of Covid-19 are never confirmed, and people who do get tested may well come disproportionately from those with symptoms of Covid-19. For these reasons, it seems plausible to assign *ψ*_*NS*_ for carriers without symptoms a normal distribution with a low mean and modest standard deviation. As approximate parameters for that distribution, we advance a mean of 0.3 and standard deviation of 0.05. (Recall that *ψ*_*NS*_ = 0.3 implies a 70% probability of going ahead with travel.)

Some potential travelers with mildly symptomatic Covid-19 will get themselves tested, and those with positive tests would not be expected to fly. But among potential passengers who are not tested and who have mild symptoms that are hard to distinguish from those of a common cold, a fraction of travelers will go ahead with their flights. We assign *ψ*_*MS*_ a normal distribution with mean 0.6 and a standard deviation .05.

One would expect that few individuals with severe/critical Covid-19 would board US flights. But “few” is not the same as none. In late 2020, two people are known to have died of Covid-19 on US domestic flights. We assign *ψ*_*CS*_ a normal distribution with mean 0.9 and a standard deviation of 0.033 (also imposing an upper limit of one).

### Distribution of ω (drop in risk with distance)

As noted, the meta-analysis in [5] estimated that viral transmission risk declines exponentially with greater distance from the contagious person. The paper’s point estimate was that risk fell by a factor of 2.02 (equivalently, risk was multiplied by 1/2.02) for each additional meter of separation, with a 95% confidence interval extending from a factor of 1.08 to one of 3.76. This interval—from roughly half the point estimate to double it—is strongly suggestive of a lognormal distribution. We chose such a distribution, and chose its parameters to match the three statistics above (1.08, 2.02, 3.71) Given that ln(1/2.02) = −.703,

ln(1/1.08) = −.077, and ln(1/3.76) = −1.324, we assign the normally-distributed *logarithm* of the decay parameter *ω* a mean of −.703 and a standard deviation of .318. (The latter statistic is (1/1.96) times the average of 1.324–.703 = .621 and .703–.077 = .626.) For those parameters, *ω* itself is assigned a mean of .521 and a standard deviation of .170.

But what of the distance d to which *ω* would be applied? In economy class in a typical Boeing 737 or Airbus 320 jet, the seats are approximately 18 in. wide while the aisle width is about 30 in.. The seatbacks in consecutive rows are separated by about 31 in.. As noted, we tie viral transmission risk to grid distances between seats. On these aircraft, grid distances on these aircraft in inches from people within three rows of a contagious person in seat 16A are:

**Table:** 

	Seat					
Row	A	B	C	D	E	F
13	93	111	129	159	177	195
14	62	80	98	128	146	164
15	31	49	67	97	115	133
16	0	18	36	66	84	102
17	31	49	67	97	115	133
18	62	80	98	128	146	164
19	93	111	129	159	177	195

Similar charts arise when the contagious passenger is in a B or C seat and, by symmetry, in the D, E, or F seats.

### The parameter λ (seatbacks as transmission barriers)

Reflecting considerable uncertainty about the benefits of seat backs, we assign *λ*--the probability that a row of seatbacks blocks viral transmissions to a preceding or following row of passengers--a normal distribution with a mean of 0.5 and a standard deviation of 0.1. Then 1 - *λ* is the failure rate of one row of seatbacks to prevent such transmission.

Passengers three or more rows from the contagious person are separated from her by a minimum of 93 in. and three seatbacks. With the distributions we use for *ω and λ*, transmission risk is so attenuated under three-row separation that we treat it as a second-order effect. Thus, we effectively adopt the two-row rule that, as noted earlier, has been advanced by the World Health Organization and the CDC.

### Distribution of *p*_*masks*_(benefit of masks)

Some passengers on US domestic flights used cloth masks over the study period, while others wear surgical masks. (Powerful N95 or KN95 masks were not generally available during that period.) The meta-analysis in [5] estimates that wearing of cloth or surgical masks reduces by an estimated 67% the chance that a viral infection is successfully transmitted. A study from China [41] about Covid-19 yields the estimate that, when everyone wears such masks, overall infection risk drops 79%. Another paper about viral infections [33] estimates that universal cloth-mask wearing would cut transmissions by 75% while surgical masks would cut transmission by 94% .

Giving by far the greatest weight to the meta-analysis, we approximate the risk multiplier *p*_*masks*_ as approximately normal with a mean of 0.3 (i.e., a 70% reduction) and a standard deviation of 0.075.

### The parameter τ_0_ (transmission risk per minute)

The estimation of *τ*_0_ − the transmission risk per minute of exposure zero distance from the contagious passenger and with no masks--is challenging. One might assume that *τ*_0_ can vary from flight to flight, because a contagious passenger who speaks and sneezes throughout the trip might cause more infections than another who sleeps all flight long. Here we rely on several research papers about actual in-flight transmissions of Covid-19 ([19–27]), the results of which are summarized in Table 2. While these papers all concern jet flights outside the United States, the flights had functioning HEPA air filters similar to those on US jets. As we discuss in the Appendix, we assign *τ*_0_ a beta distribution with parameters *α* = 1 and *β* = 520.

**Table 2:** Some details about ten jet flights on which some boarding passengers carried Covid-19 (Sources: References [19–27])

Routing	Duration(Hours)	Boarding passengers with infections.	% of Nearby passengers infected in flight^1^	Masks?
London-Hanoi	10.2	1	92% (11/12)	No
Tel Aviv-Frankfurt	4.7	7	17% (2/12)	No
Sydney-Perth	5.0	11	40% (8/20)	No
Osaka-Okinawa	2.0	1	30% (7/23)	Some^2^
Guangzhou-Toronto	15.1	2	0%	Yes
Dubai-Auckland	15.0	2	50% (4/8)	Yes
Dubai-Dublin	7.8.	9^3^	40% (4/10)	Yes
Singapore-Hangzhou	5.0	15	2% (1/50)	Yes
Milan-Seoul	11.0	6	3% (1/38)	Yes^4^
Milan-Seoul	11.0	3	Unclear	Yes^5^

^1^Based on initially-uninfected passengers within two rows of at least one contagious passenger

^2^Infected passenger wore no mask, but most other passengers did

^3^Journal article states that “at least four” of the thirteen passengers who tested positive for Covid-19 after the flight contracted the disease on board

^4^Passengers wore extremely effective N95 masks; on-board infection is suspected to have occurred in lavatory

^5^Passengers wore N95 masks; one on-board infection but seating location not reported

### Estimation of *P*_*ASYM*_, *P*_*PRESYM*_, *P*_*MM*_, and *P*_*SC*_

We follow a different approach in estimating the parameters about the course of contagious Covid-19, namely, *P*_*ASYM*_, *P*_*PRESYM*_, *P*_*MM*_, and *P*_*SC*_. Consistent with available literature [28–32], we treat current carriers of Covid-19 as having become infected over the last seven days, and assume that about 30% of them are asymptomatic. Among the 70% infected in the last week who will develop symptoms, we use the estimate that those infected in the last two days (i.e, approximately 2/7 of the group) are pre-symptomatic, while the remaining 5/7 show symptoms of varying severity. Similarly, we work with published estimates that approximately 4/5 of symptomatic carriers will develop mild/moderate symptoms, while 1/5 will have severe/critical ones. For these reasons, we make the approximations:

$${\displaystyle \begin{array}{cc}{P}_{ASYM}\approx 0.3& {P}_{PRESYM}\approx 0.7\ast \left(\frac{2}{7}\right)=0.2\\ {}{P}_{MM}\kern0.5em \approx 0.7\ast \left(\frac{5}{7}\right)\ast 0,8=0.4& {P}_{SC}\approx 0.7\ast \left(\frac{5}{7}\right)\ast 0.2=0.1\end{array}}$$

In the analysis, we worked with these point estimates of Covid-19 infection parameters, rather than with probability distributions. We did so in the belief that adding several more distributions to the modeling would do more to make the formulation unwieldy than to offer greater insight about Covid-19 transmission on airplanes.

## Results

(i) *Individual Flights*

Given the number of random variables in the expression for P_N,X_ and their differing distributions, the distribution for P_N,X_ does not take any familiar analytic form. It is necessary to use simulation to investigate the behavior of P_N,X_ and we did so, treating different random variables as independent. In most respects, an independence assumption is highly plausible: the uncertainty about the “healthy traveler” parameter seems unrelated to uncertainty in the effectiveness of seatbacks.

We conducted 10,000 simulations for each month from June 2020 to February 2021, assuming an all-economy configuration of the 737 or 320 aircraft with 29 rows and 174 seats. We considered six possible occupancy levels for the seats:

- All seats full
- 5/6 of seats full, with half the middle seats empty but all other seats occupied
- 2/3 of seats full, including all window and aisle seats occupied but none in the middle
- Half of seats full, consisting of three in each row scattered among the four window and aisle seats
- 1/3 of seats full, consisting of two in each row, one on each side of the aisle in the window or aisle position
- 1/6 of seats full, consisting of one window or one aisle seat in each row

Table 3 presents various percentiles for the probability that an uninfected passenger would contract Covid-19 during a two-hour flight on an “average” day over June 2020–February 2021. (i.e., one for which (N_7_ /N) was at the average for the period). The table reports on three possibilities: the flight was full, 2/3 full, or 1/3 full. In all of the columns, the distribution for risk has high variability, with the 10th percentile about 1/7 of the median and the 90th percentile nearly four times the median. Such variability was unavoidable because several of the “input” distributions in the simulation had high coefficients of variation.

**Table 3:** Estimated distribution for Covid-19 infection probability for randomly-chosen uninfected travelers on November 1, 2020, for two hour US domestic jet flights on Boeing 737 or Airbus 320, at three levels of seat occupancy

	Full	2/3 Full	1/3 Full
10th Percentile	1 in 9050	1 in 14,900	1 in 39,700
25th Percentile	1 in 3350	1 in 5550	1 in 14,350
50th Percentile	1 in 1350	1 in 2200	1 in 5550
75th Percentile	1 in 650	1 in 1050	1 in 2600
90th Percentile	1 in 350	1 in 600	1 in 1450
Mean	1 in 900	1 in 1400	1 in 3500
Standard Deviation	1 in 850	1 in 1350	1 in 3150

Table 4 presents mean and median risk at all six levels of crowding that were considered. (Rather than designate the mean or the median of the simulation results as the point estimate for infection risk, we give equal prominence to both.) The mean and the median risk estimates diverge somewhat, presumably because several distributions for the parameters had long right tails. But both the mean and median suggest a risk around 1 in 1000 of contracting Covid-19 in flight on a full plane on an average day. The table also indicates that, as the flight becomes less full, infection risk drops more than proportionately. For example, the mean risk estimate on a 55% full flight is not 55% of that for a full flight, but instead 46% of it. If the flight is 55% full, then its expected number of contagious passengers is 55% as high as that for a full flight. But the uninfected passengers are on average further away from the contagious one (especially because the seat next to that traveler is empty). For that reason, risk declines not by 45% but by 54%.

**Table 4:** Mean and median estimates of Covid-19 infection probability for two hour US domestic jet flights on Boeing 737 or Airbus 320 on November 1, 2020, at six levels of seat occupancy

Seat occupancy	Mean	Median
100%	1 in 900	1 in 1350
83.33%	1 in 1100	1 in 1650
66.67%	1 in 1400	1 in 2200
50%	1 in 2150	1 in 3350
33.33%	1 in 3500	1 in 5550
16.67%	1 in 9950	1 in 16,700

Tables 5 and 6 elaborate on this last point by offering estimates of the probability of becoming infected during a two-hour flight given a contagious traveler is in seat 16A, as a function of the uninfected traveler’s seat in rows 14–18 (and assuming mask usage). Under the “two row” approximation, passengers in other rows are assigned infection probabilities of zero.

**Table 5:**
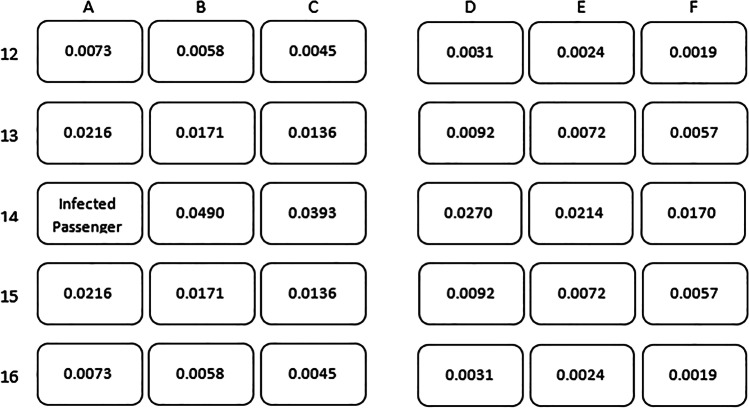
Estimated risk of contracting Covid-19 on two-hour US domestic jet flights on Boeing 737 or Airbus 320 for each seat position, given that the flight is full and a single contagious passenger is sitting in Seat 14A

Average Risk for 29 Passengers within Two Rows of Contagious One: 1 in 82

Average Risk for all 173 Uninfected Passengers (which is zero beyond Two Rows from Contagious One): 1 in 491

**Table 6:**
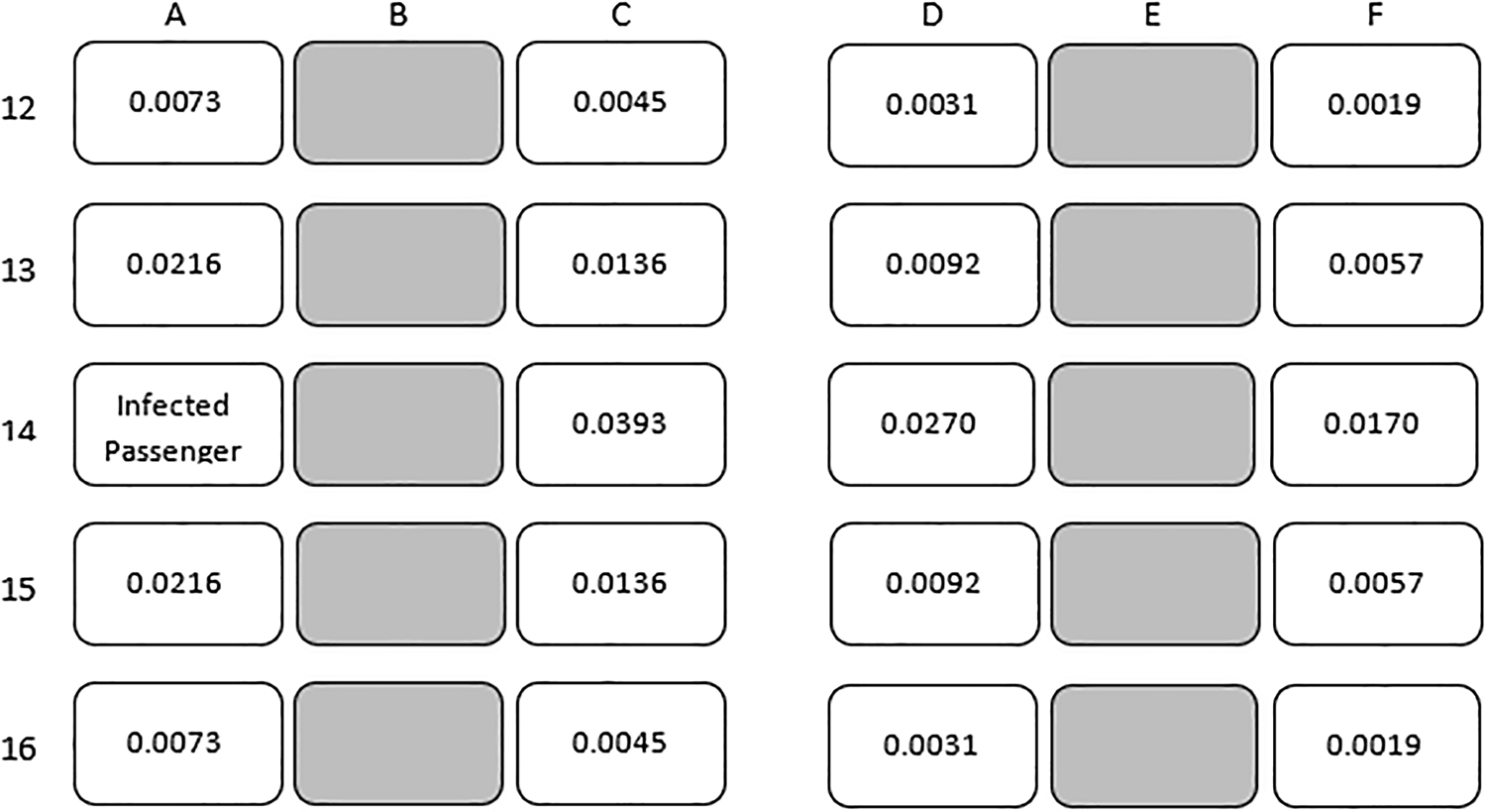
Estimated risk of contracting Covid-19 on a two hour US domestic jet flights on Boeing 737 or Airbus 320 for each seat position, given that all of the middle seats on the flight are vacant and a single contagious passenger is sitting in Seat 14A

Average Risk for 19 Passengers within Two Rows of Contagious One: 1 in 86

Average Risk for all 115 Uninfected Passengers (which is Zero beyond Two Rows from Contagious One): 1 in 526

**Fig. 2:**
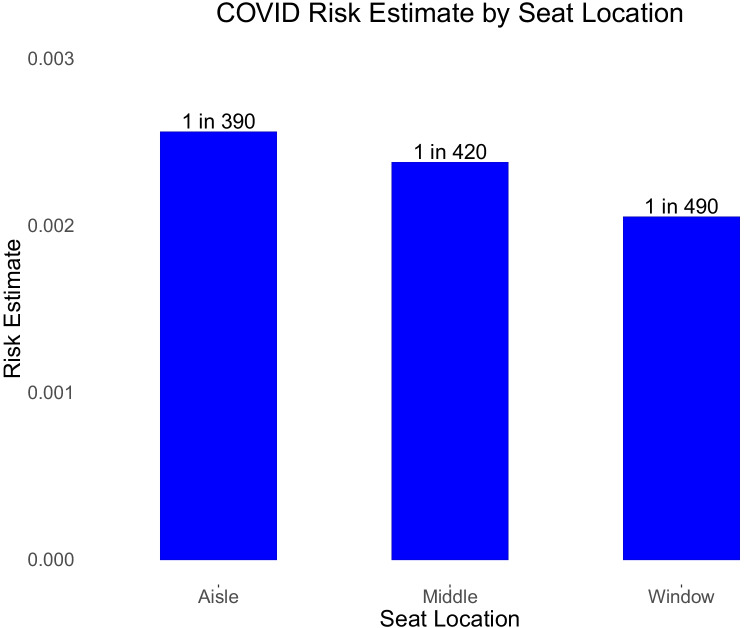
Probability of contracting Covid-19 on two hour domestic US jet flight on 11/1/20, based on type of seat of single contagious passenger on fully-loaded Boeing 737 or Airbus 320

Fig. 2 shows that the contagious passenger infects more other travelers if she is in an aisle seat than in a middle seat, while the middle seat yields more infections than a window seat. However, the effect is not especially large.

(ii) *Flights over the Nine-Month Period*

So far, we have considered the general question: if p_CONTAG_ is the probability that each boarding passenger has contagious Covid-19 and N is the total number of passengers on the plane, what is the probability that an uninfected passenger will contract Covid-19 in flight? But we have not considered what proportions of actual passengers faced which specific (N, p_CONTAG_) combinations. Here we try to address this issue by merging the existing “what if?” analysis with data about the ebbs and flows of the Covid-19 pandemic over the nine months of interest, as well as actual seat occupancy rates on US jets during that period.

We are fortunate to have obtained data about the distribution of seat occupancy by for US airlines month from June 2020 to February 2021, broken down by deciles. We can use these data in estimating overall infection risk if we make two assumptions (i) that average occupancy level within a decile is at the middle of that decile (e.g., 55% for the range 50%–60%) and (ii) the distribution of occupancy on 737 and 320 flights is about the same as the distribution over all US flights.

Table 7 offers seat-occupancy data for the month November 2020, along with a tabulation of infection risk for each occupancy range as a multiple of the risk for a full flight.

**Table 7:** Some details about US domestic jet flights in November 2020, related to the risk of getting infected with Covid-19 on board

Seat occupancy range	Estimated % of seats filled	November 2020% of US flights	Infection risk relative to full plane	
			Mean	Median
0’s	0.05	1.2	0.03	0.02
10’s	0.15	3.5	0.08	0.07
20’s	0.25	6.6	0.17	0.16
30’s	0.35	11.1	0.27	0.26
40’s	0.45	14.7	0.36	0.35
50’s	0.55	18.9	0.47	0.46
60’s	0.65	18.2	0.60	0.59
70’s	0.75	7.6	0.71	0.71
80’s	0.85	8.7	0.83	0.83
90’s	0.95	9.6	0.94	0.94

To move from this information to infection risk for travelers, we work with eq. (2). Doing so properly reflects the points that a plane with 174 seats that is 80% full carries four times as many passengers as another such plane that is 20% full .

Table 8 presents mean and median estimates of infection risk by month for randomly-chosen travelers of 737 or 320 jets. The risk estimates vary considerably over time, and are highest for January 2021 when rates of new Covid-19 infections in the US were surging. Both the mean and median risk estimates are 1 in 2250 or higher for the full period. Not everyone would agree with Southwest Airlines that this level of risk is “virtually nonexistent.”

**Table 8:** Mean and median estimates of risk that randomly-chosen uninfected traveler contracts Covid-19 on board US Boeing 737 or Airbus 320, by month from June 2020 to February 2021

Month	Total US airline passengers^1^(Millions)	Mean on-board infection risk^2^	Median on-board infection risk
June 2020	14.5	1 in 3250	1 in 5000
July 2020	20.7	1 in 3300	1 in 5050
August 2020	21.7	1 in 3950	1 in 6050
September 2020	21.5	1 in 3150	1 in 4850
October 2020	25.6	1 in 1750	1 in 2700
November 2020	25.5	1 in 1050	1 in 1600
December 2020	26.4	1 in 850	1 in 1300
January 2021	23.6	1 in 900	1 in 1400
February 2021	24.4	1 in 1650	1 in 2500
Total	204.0	1 in 1450	1 in 2250

^1^Based on passengers processed at US airports by Transportation Security Administration (TSA), which includes a very small proportion of international travelers. We treat the effect of international passengers on TSA counts as second-order

^2^Arithmetic average of estimated risks for all uninfected passengers that month, taking into account the likelihood that a contagious traveler is on board the flight, the effectiveness of masks, the distribution of seat-occupancy levels, and the relative locations of contagious traveler and uninfected ones

It is natural to consider how many US deaths resulted from Covid-19 infections contracted on airplanes. But estimating that number is extremely difficult. Moreover, a substantial fraction of the deaths ultimately caused by on-board Covid-19 infections could befall people who were never on the plane.

One can make rudimentary calculations tied to the estimate that 1 in 2000 uninfected air travelers contracted Covid-19 on US domestic flights over June 2020 to February 2021. Given 204 million passengers in total, the 1 in 2000 rate implies about 100,000 infections in total. If one believes that every 1000 on-board infections ultimately led to one death, then the death toll would be 100. For one death per 500 on-board infections, the corresponding toll would be 200; for one death per 100 such infections, the death toll would reach 1000. While such numbers fall far below the hundreds of thousands of US deaths in the pandemic, even 100 fatalities over a nine-month period compares unfavorably to the average of one passenger death every nine months on US flights over 2010–19.

## Some other assessments of in-flight Covid-19 transmission risk

As noted, two major studies about on-board transmission of Covid-19 on airplanes did not use data about actual passengers. Here we discuss them briefly.

The Aviation Public Health Initiative of the Harvard School of Public Health extensively studied the risk of Covid-19 transmission during air travel [42]. The study offered many good practical suggestions about flying during the pandemic, but only limited guidance about the magnitude of on-board infection risk. Its overall assessment was the risk is lower than that “in indoor restaurants or grocery stores,” but these were known to be especially hazardous venues during the pandemic. Moreover, HSPH’s assertion that on-board ventilation systems “effectively counter the proximity travelers will be subject to during flights.” was an overstatement given the evidence in Table 3.

The US Department of Defense [43] conducted an imaginative study about viral transmissions of disease on aircraft. It used mannequins wearing surgical masks to simulate coughing passengers and used aerosol tracers to see where the emitted particles went. However, the mannequins in the study faced forward and did not “speak” or move, while possible transmission via droplets was not considered. The researchers suggested that on-board infection risk was low but offered no numerical estimate.

## Final remarks

We readily acknowledge that this analysis is imperfect, but hope that its strengths push it to the positive side of the ledger. It seemed worthwhile to use various data sets to estimate the prevalence of contagious Covid-19 among Americans who boarded airplanes over June 2020 to February 2021 It seemed sensible to use peer-reviewed reports about Covid-19 transmission on airplanes as guides to the likelihood that on-board-infections will occur. And it seemed desirable to work with actual seat-occupancy data, so that the modeling could assess the experiences of typical passengers, and not just worst-case scenarios like full flights at the height of the pandemic.

The situation presumably changed with the widespread availability of vaccines starting in early 2021, but also with the sharp increase in contagiousness of Covid-19 with the arrival of the Delta and Omicron variants. Modeling like that presented here could help in assessing the changed situation, much as the general approach might help in connection with a future pandemic. And that pandemic might arise sooner than any of us would like.

### Supplementary Information

ESM 1 (CSV 5 kb)

ESM 2 (CSV 5591 kb)

ESM 3 (IPYNB 91 kb)

ESM 4 (CSV 4 kb)

## Appendix: Estimation of the parameter

For individual flights discussed in the literature, an estimate of π_0_ can arise from data about flight duration, the number of contagious passengers, and the fraction of passengers within two rows of contagious travelers who became infected in flight. Table 3 presents the particulars for ten flights during the Covid-19 pandemic that were discussed in peer-reviewed publications. These international flights are all those mentioned in the review paper [18] that provide information about where the contagious passenger(s) were seated, and which passengers within two rows of them are believed to have contracted Covid-19 during the flight

Using mid-range estimates for *ω and λ*,estimating π_0_ is most straightforward on flights on which no one wore masks. Yet the estimates arising from such flights exhibit large variation. On the five-hour Sydney-Perth flight, the π_0_−estimate is about .0015 per minute in the air. For the Tel Aviv-Frankfurt flight on which seven infected passengers were seated together, the corresponding estimate is about .0005. Yet the 92% rate of nearby secondary infections on Vietnam Flight 54 implies a π_0_ -value of .005 if not higher. So does the 30% rate on the short flight from Osaka to Okinawa.

Flights on which passengers wore masks are less helpful in estimating π_0_, which assumes the absence of masks. The scarcity of infections on the Guangzhou-Toronto and Italy-Korea flights offers little reliable information about π_0_. On the first of these flights, there were no transmissions, but the two contagious passengers and all other passengers wore masks. On the other two flights, passengers wore powerful N95 masks and one on-board infection occurred. Thus, even if π_0_ were high, mask usage could well explain the paucity of transmission on these flights.

Still, we can try to extract some information about π_0_ from flights on which masks were worn. If masks are treated as 70% effective, then a crude rule would assume that the observed number of in-flight infections was about 30% as high it would have been without masks. Applying this principle for Singapore-Hangzhou yields π_0_ ≈ .0001 . Reluctantly applying it to Guangzhou-Toronto would yield π_0_ ≈ 0 . On the Dubai-Auckland flight, 50% of the passengers near a contagious traveler contacted secondary infections despite the use of masks. Yielding .002 is a lower bound for π_0_ based on that flight. On the flight to Ireland, 40% of travelers seated near infected passengers got secondary infections during the seven-hour flight Here the lower bound for π_0_ is .001. These eight π_0_ -estimates arising from the published literature—namely 0, .0001, .001+ .0005, .0015, .002+, .005+ and .005 + −--are individually imprecise, and in any case constitute a small sample. If we treat .001+, .002+ and .005+ as, respectively, .001 and .002 and .005 we can assign π_0_ a beta distribution with parameters *α* = 1 and *β* = 520. This beta distribution matches the mean and standard deviation of the eight numbers above (.0019 and .0019).

## Glossary of Variables

P: Probability that a randomly chosen uninfected-traveler among those under study contracts COVID- 19 in flight
P_N,X_: *P*(randomly chosen uninfected passenger boards a flight with *N*, X of whom board harboring Covid-19)
Q_N,X_: Conditional probability that a random uninfected passenger contracts COVID-19 on board, given N and X
X: Number of contagious passengers on board a flight of interest
N: Total Number of passengers on the flight
M: Number of non-stop US flights over an observation period on a 737/A320 aircraft (174 seats)
q_Z_: Fraction of flights of interest with Z passengers on board
Q_Z_: Probability a random air traveler over the period of interest boards a flight with a total of Z passengers (i.e., P(N = Z))
P_CONTAG_: Probability that a randomly chosen passenger who boards a flight carries COVID-19
γ: Fraction of potential US air travelers who carry COVID-19
C_7_: Number of confirmed cases of COVID-19 in the US over the past 7 days
POP: Population of the US
ρ: multiplier to adjust for the extent to which confirmed COVID-19 cases in the US underestimate the actual number of cases
β: “healthy passenger” factor to reflect the fact that US air travelers come disproportionately from communities where COVID-19 is less common than in the population at large
η: Fraction of COVID-19 cases that end in death
N_D_(t): Number of new US COVID-19 cases on day t that eventually ended in death
D(t + j): Recorded number of US COVID-19 deaths on day t + j
R_CV_: Per capita rate of confirmed Covid-19 infections in the overall population
R_i_: Per capita rate of confirmed Covid-19 infections in communities in median income range i
I_i_: Fraction of citizens living in communities in median income range i who
n: Number of median income ranges
T_i_: Annual air trips per capita in communities in median income range i
R_CVT_: Probability of having COVID-19 adjusted for communities in income ranges with disproportionate shares of air travelers
α: Generic Probability that a potential air traveler not suffering from COVID-19 would fly
f: Overall probability that someone infected with COVID-19 who would otherwise fly will not do so
P_ASYM_: Probability that someone contagious with COVID is asymptomatic
P_PRESYM_: Probability that someone infected with COVID is pre-symptomatic at a random time while contagious
P_MM_: Probability that someone infected with COVID-19 has mild/moderate symptoms at a random time while contagious
P_SC_: Probability that someone infected with COVID-19 has severe/critical symptoms at a random time while contagious
Ψ_NS_: Conditional probability that an asymptomatic/pre-symptomatic COVID-19 carrier who would otherwise fly will not do so
Ψ_MS_: Conditional probability that a COVID-19 carrier with mild/moderate symptoms who would otherwise fly will not do so
Ψ_CS_: Conditional probability that a COVID-19 carrier with severe/critical symptoms who would otherwise fly will not do so
V_S1,S2_: Risk per minute that a passenger from Seat S_2_ would contract COVID-19 from a passenger in Seat S_1_
d: Grid distance between Seat S_1_ and Seat S_2_
ω: Rate of exponential decay of transmission risk with distance, assuming no barriers
λ: Probability that an individual seatback blocks transmission from a contagious passenger
π_0_: Level of infection risk per minute at distance zero from contagious Covid-19 passenger
R: Number of rows between Seat S_1_ and Seat S_2_
p_masks_: Probability that universal masking blocks a transmission of COVID-19 from an infected traveler to an uninfected one
Z_S1,S2_: Probability that COVID-19 is transmitted during the flight from a contagious passenger in Seat S_1_ to an uninfected passenger in Seat S_2_
T: Duration of the flight
U(S_1_): Probability that a randomly-chosen uninfected passenger on a flight contracts COVID-19 from the passenger in Seat S_1_
